# Correction: Highly efficient green up-conversion emission from fluoroindate glass nanoparticles functionalized with a biocompatible polymer

**DOI:** 10.1039/d2ra90077g

**Published:** 2022-08-12

**Authors:** G. Lesly Jimenez, Binita Shrestha, Tyrone Porter, Bartlomiej Starzyk, Magdalena Lesniak, Marta Kuwik, Marcin Kochanowicz, Magdalena Szumera, R. Lisiecki, D. Dorosz

**Affiliations:** Faculty of Materials Science and Ceramics, AGH University of Science and Technology A. Mickiewicza 30 30-059 Krakow Poland glesly@agh.edu.pl; The University of Texas at Austin Austin 78-712 Texas USA; Institute of Chemistry, University of Silesia Szkolna 9 40-007 Katowice Poland; Faculty of Electrical Engineering, Bialystok University of Technology Wiejska 45D Street 15-351 Bialystok Poland; Optical Spectroscopy Division, University of Wrocław plac Uniwersytecki 1 50-137 Wrocław Poland

## Abstract

Correction for ‘Highly efficient green up-conversion emission from fluoroindate glass nanoparticles functionalized with a biocompatible polymer’ by G. Lesly Jimenez *et al.*, *RSC Adv.*, 2022, **12**, 20074–20079, https://doi.org/10.1039/D2RA03171J.

The authors regret that the name of one of the authors (Tyrone Porter) was shown incorrectly in the original article. The corrected author list is as shown above.

The Royal Society of Chemistry apologises for these errors and any consequent inconvenience to authors and readers.

## Supplementary Material

